# One-carbon metabolism and nucleotide biosynthesis as attractive targets for anticancer therapy

**DOI:** 10.18632/oncotarget.15053

**Published:** 2017-02-03

**Authors:** Oleg Shuvalov, Alexey Petukhov, Alexandra Daks, Olga Fedorova, Elena Vasileva, Nickolai A. Barlev

**Affiliations:** ^1^ Institute of Cytology RAS, Saint-Petersburg, Russian Federation; ^2^ Institute of Hematology, Almazov Federal North-West Medical Research Centre, Saint-Petersburg, Russian Federation

**Keywords:** cancer metabolism, one-carbon metabolism, anti-cancer therapy, c-Myc, p53

## Abstract

Cancer-related metabolism has recently emerged as one of the “hallmarks of cancer”. It has several important features, including altered metabolism of glucose and glutamine. Importantly, altered cancer metabolism connects different biochemical pathways into the one fine-tuned metabolic network, which stimulates high proliferation rates and plasticity to malignant cells. Among the keystones of cancer metabolism are one-carbon metabolism and nucleotide biosynthesis, which provide building blocks to anabolic reactions. Accordingly, the importance of these metabolic pathways for anticancer therapy has well been documented by more than fifty years of clinical use of specific metabolic inhibitors – methotrexate and nucleotides analogs. In this review we discuss one-carbon metabolism and nucleotide biosynthesis as common and specific features of many, if not all, tumors. The key enzymes involved in these pathways also represent promising anti-cancer therapeutic targets. We review different aspects of these metabolic pathways including their biochemistry, compartmentalization and expression of the key enzymes and their regulation at different levels. We also discuss the effects of known inhibitors of these pathways as well as the recent data on other enzymes of the same pathways as perspective pharmacological targets.

## INTRODUCTION

Cancer cells represent a great degree of adaptation due to the high plasticity of their genomes [1]. This plasticity is associated with genomic instability, which is the hallmark of all tumors [2]. As the result of such plasticity, cancer cells are able to rapidly acquire resistance to anti-cancer chemotherapeutics [3]. Therefore, the search for new biomarkers and the corresponding molecular processes that profoundly discriminate cancer cells from the normal ones is the subject of intense studies.

One of the fundamental features shared by all malignancies is their altered, compared to normal tissues, metabolism [4–6]. Oncogene-dependent increased proliferation of cancer cells is tightly connected with their ability to respond to nutrients and mitotic signals that coordinate uptake of nutrients and subsequent anabolism [5]. Thus, it is deemed that activation of proliferation promotes metabolic transformation.

Primarily, common cancer-related metabolic alterations include: increased uptake of the glucose and glutamine, up-regulated “aerobic glycolysis”, pentose-phosphate pathway, active one-carbon metabolism, nucleotide biosynthesis and acquired ability to *de novo* synthesize fatty acids.

One-carbon (1C) metabolism functions as a regulator and sensor of the cells’ nutrient status through cycling of 1C-groups and allocating them between different acceptor compounds. It is important to note that 1C-metabolism controls synthesis of nucleotides, certain aminoacids, S-adenosylmethionine (SAM), glutathione, and other cellular processes important for rapidly proliferating malignant cells [7]. Moreover, one-carbon metabolism can contribute to the energy balance, providing molecules of ATP and NADPH [8, 9]. Thus, 1C-metabolism not only dispenses carbon atoms between various acceptor molecules required for biosynthesis, but it also tunes cells’ nutrient status with epigenetic and redox statuses [10].

The importance of 1C-metabolism and nucleotide biosynthesis as targets for anti-cancer therapy has been proved by a more than 60-years therapeutic use of Methotrexate (MTX) and Thiopurines, inhibitors of the 1C-metabolism and nucleotide biosynthesis, respectively. Notably, the growing body of evidence suggests that these metabolic pathways should be viewed as a complex network [8, 9, 11, 12]. Moreover, up-regulation of these pathways as well as specific oncogenic features of a number of functionally related enzymes of one-carbon metabolism, including phosphoglycerate dehydrogenase PHGDH [13], phosphoserine aminotransferase PSAT1 [14], phosphoserine phosphatase PSPH [15], serine hydroxymethyltransferase SHMT2 [16], glycine dehydrogenase GLDC [17], inosine-5′-monophosphate dehydrogenase IMPDH2 [18]- became also known.

In this review, we discuss the 1C-metabolism and nucleotide biosynthesis as common and specific features of tumors, which also provide a promising therapeutic approach for specific elimination of cancer cells since they are highly sensitive to inhibition of these pathways.

## “INPUTS” OF ONE-CARBON METABOLISM

**Figure 1:**
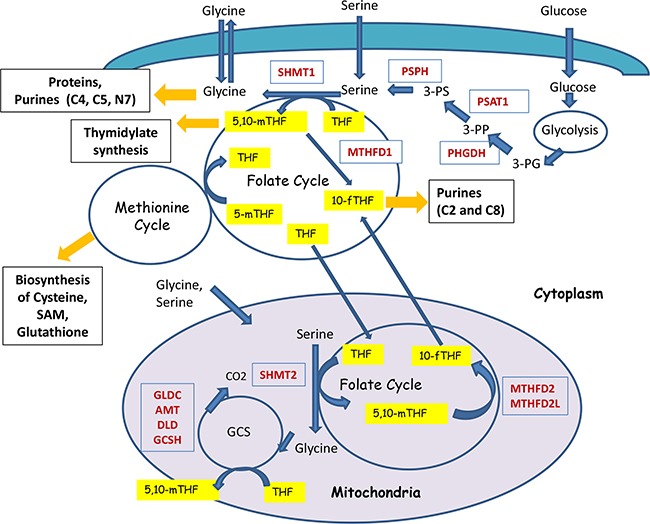
Schematic representation of the compartmentalization and enzymatic reactions of one-carbon metabolism One-carbon metabolism acts as a gauge of the cell nutrient status by redistributing carbon groups from serine and glycine, called “inputs”, to generate various compounds, called “outputs” (shown in black boxes) that serve as building blocks for cell biosynthesis. Also, they maintain the redox and methylation states of cells. Serine and Glycine can be imported through the membrane (shown as green layer) into the cells or it can be synthesized from the intermediate of glycolysis – 3-PG. Metabolic cycles are denoted as circles. Critical enzymes are shown in red. Carriers of one-carbon groups are shown in yellow. For example, 5,10-methyleneTHF provides one-carbons for thymidylate synthesis, catalyzed by the enzyme called Tymidylate Synthase. The positions of one-carbons used for the synthesis of purines (C2, C4, C5, and C8 carbons of purine rings) are indicated. Folate cycle is tightly connected with Methionine cycle. Folate cycle operates both in the cytoplasm and in mitochondria (magenta colored circle) and are linked through Tetra Hydro Folate (THF).

As mentioned above, one-carbon metabolism acts as an integrator of the cell nutrient status by redistributing carbon groups from certain aminoacids, usually serine and glycine, (called “inputs”) to generate various compounds (“outputs”) that serve as building blocks for cell biosynthesis and also maintain the redox and methylation states of cells [7]. Serine can be obtained exogenously (i.e. imported from outside of the cell) as well as endogenously by *de novo* synthesis (see details below and in Figure 1). Glycine can be also transported through the plasma membrane [16]. Alternatively, it can be generated from serine through an enzymatic conversion in either cytoplasm or mitochondria. Furthermore, glycine can also be synthesized from threonine as was shown for mouse embryonic stem cells [19].

In theory, both serine and glycine can be potential donors of 1C-groups for one-carbon metabolism. However, the actual relationship between serine and glycine metabolism is far more complex. The integrated scheme summarizing the crosstalk of serine and glycine metabolic pathways is presented in Figure 1.

### Serine

There are evidences that cancer cells usually demonstrate increased serine and glycine biosynthesis and uptake [13, 16, 20, 21]. *De novo* serine synthesis consists of three steps and involves the conversion of 3-phosphoglycerate (3-PG, an intermediate of glycolysis) to 3-phosphopyruvate (3-PP) by the Phosphoglycerate Dehydrogenase (PHGDH) (Figure 1). The next step involves conversion of 3-PP to 3-phosphoserine (3-PS) which is mediated by the Phosphoserine Aminotransferase (PSAT1) using glutamate for this transamination. As the final step, the phosphate ester is hydrolyzed by the Phosphoserine Phosphatase (PSPH), resulting in production of serine. Apparently, different cancer cells promote expression of the corresponding enzymes to increase the biosynthesis of serine [13, 21, 22].

It has been shown that cancer cells utilize up to 10% of glycolytic intermediate 3-PG for serine biosynthesis [13]. PHGDH is amplified in a number of cancers, including 6% of breast cancers and 40% of melanomas [21]. Moreover, experiments using siRNA demonstrated that attenuation of PHGDH expression was associated with slow cell growth of non-malignant cells. On the contrary, ectopic expression of PHGDH in the non-cancerous MCF10A breast epithelial cell line disrupted acinar morphogenesis and induced other phenotypic alterations that may predispose cells to transformation [13].

There are also evidences of de-regulated expression in cancer of two other enzymes of serine biosynthesis – PSAT1 [14, 23, 24] and PSPH [15, 22].

Besides serine, which represents a critically important “input” of one-carbon metabolism and nucleotide biosynthesis, there is another important metabolite generated at the transamination step of serine biosynthesis - α-ketoglutarate (αKG) [25]. αKG is the entry point through which glutamine supplies carbon to the tricarboxylic acid (TCA) cycle during cell growth, enabling the production of a number of essential biosynthetic precursors [26].

Taken these results together, it is evident that serine is a hub of one-carbon metabolism and therefore its overexpression is an important feature of different malignancies.

### Glycine

Whereas the importance of serine for the enhanced proliferation of cancer cells is generally accepted, the impact of glycine on this process is the topic for intense debates.

Jain with colleagues [16] applied a mass-spectrometry approach to measure the consumption and release of 219 metabolites from the media across the NCI-60 panel of cancer cell lines and combined these data with the pre-existing atlas of gene expression. The integrated analysis identified glycine consumption as well as expression of the mitochondrial glycine biosynthetic pathway (SHMT2, MTHFD2 and MTHFD1L) to be strongly correlated with the rates of proliferation across all cancer cell lines.

However, other works have shown that cancer cells fail to consume glycine when serine is abundant [20, 27]. For instance, Labuschange (2014) [27] showed that cancer cells preferentially consumed serine rather than glycine, and the high level of serine uptake paralleled with glycine efflux. Moreover, according to their results, the excess of glycine even inhibited cell growth. Biochemically, high levels of glycine inhibited the metabolic transformation of the former into purines, required for DNA replication, by driving instead the intracellular glycine-to-serine conversion. Glycine was converted to serine at the expense of 5,10-methylenTHF, thus depleting its intracellular pool and hence slowing the cell growth. Based on these data, it has been proposed that cancer cells release the excess of glycine thereby limiting its intracellular concentration to facilitate serine uptake and serine-to-glycine conversion [28]. Notably, it is the serine-to-glycine conversion process that yields the 5,10-methyleneTHF metabolite required for the maintenance of maximal levels of nucleotide synthesis and proliferation. Serine is converted to glycine by two isoforms of Serine hydroxymethyltransferase (SHMT1 and SHMT2) that correspond to the cytosolic and mitochondrial forms, respectively. The excess amount of glycine, the product of this reaction, can reverse this reaction and therefore should be removed from the cell [27]. On the related note is the fact that two different cancer *in vivo* models also demonstrated that excess of dietary glycine inhibited the development of tumors [29, 30]. Collectively, glycine biosynthesis is deemed as the central process which sustains one-carbon metabolism and rapid proliferation.

As mentioned above, glycine metabolism is intimately linked with purine biosynthesis and defines the sensitivity to mycophenolate, tiazofurin, alanosine and other inhibitors of purine biosynthesis [16]. In general, glycine can contribute to purine biosynthesis in two ways: by direct incorporation into the purine ring or indirectly, by providing one-carbon units for biochemical reactions involved in the purine ring biosynthesis. The latter can be derived by either synthesis of glycine from serine (the SHMT-catalyzed reaction), or alternatively, by glycine degradation (oxidization to CO2 by a highly evolutionary conserved glycine cleavage system - GCS) (Figure 1). It is important to note that the incorporation of glycine into purine nucleotides does not involve oxidation by GCS but is rather mediated by SHMT-derived glycine [16]. These data suggest that SHMT-catalyzed glycine synthesis together with direct incorporation of glycine into the purine rings link glycine production with purine biosynthesis.

As mentioned above, the glycine degradation is exerted by GCS. This system consists of four mitochondrial proteins: the T-protein (GCST or AMT (aminomethyltransferase)), P-protein (GLDC (glycine dehydrogenase)), L-protein (GCSL or DLD (dihydrolipoyl dehydrogenase)), and H-protein (GCSH). It converts glycine to CO2 in the following reaction: Glycine + THF + NAD^+^ = 5,10-methylene-THF + CO_2_ + NH_3_ + NADH_2_ [31]. Importantly, the products of this enzymatic reaction, 5,10-methyleneTHF and NADH, are required for the nucleotide biosynthesis.

Apparently, GCS is critical for efficient elimination of the excess of glycine. Components of GCS, especially GLDC, are frequently overexpressed in different malignancies and this is linked with cancer progression. Tedeschi and colleagues [9] showed that about 28% of lung-, 19% of breast-, 9% of prostate-, 30% of colorectal-, 23% of brain- and 21% of ovarian cancers exhibit a significant up-regulation of the 1C-metabolism gene signature, including GLDC. The data from other groups support the notion on overexpression of GCS components in cancer [12, 16].

Zhang and colleagues [17] have established GLDC as instrumental for the growth and tumorigenesis of tumor-initiating cells derived from the primary NSCLC. Overexpression of GLDC promotes cellular transformation and induces dramatic changes in glycolysis and serine/glycine metabolism, leading to changes in pyrimidine metabolism and cancer cell proliferation. Furthermore, its aberrant regulation often correlates with poor survival of lung cancer patients.

Another study [32] has shown that GCS may cooperate with SHMT2 to ensure survival and progression of tumors. For example, GLDC-mediated cleavage of the excessive amount of glycine supported the growth of glioma cells with active SHMT2 under ischemic conditions. On the contrary, when the activity of GLDC was inhibited, cells with high levels of SHMT2 were selectively killed. This is explained by the fact that the excess of glycine generated by SHMT2 was subsequently converted into two toxic molecules, aminoacetone and methylglyoxal, which normally are metabolized by GLDC [32].

Taken these data together, one can reckon that despite the fact that glycine biosynthesis is the hub of one-carbon metabolism, the excess of glycine itself can be detrimental to the tumor cell proliferation and needs to be strictly controlled by either its export or by GCS-mediated clearance.

## FOLATE AND METHIONINE CYCLES

The “core” part of the one-carbon metabolism comprises the Folate and Methionine cycles, which are linked together. These two cycles integrate cell nutrient status using 1C-groups from glycine and serine as “inputs” to generate different “outputs” such as nucleotides, glutathione, SAM, and other metabolites, which are required for DNA and RNA biosynthesis, as well as for the maintenance of the redox and epigenetic cell states.

### Folate cycle

**Figure 2:**
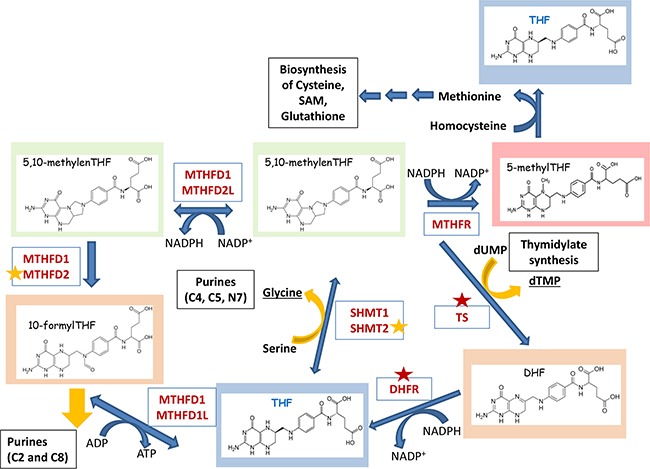
Folate cycle, its “outputs” and the energy balance Critical enzymes of the Folate cycle are shown. TetraHydroFolate (THF) is a carrier that distributes one-carbon groups (1C-group) from serine to different “outputs” – thymidylates, purines, SAM, GSH, etc (shown in black boxes). After accepting the 1C-group, THF undergoes modifications that alter its oxidation states: 10-formylTHF, 5,10-methyleneTHF, 5-methylTHF (shown in different background colors). Donated carbon and nitrogen atoms corresponding to their numbers in the pyrimidine and purine rings are shown in brackets. Red asterisks indicate the enzymes that are currently being explored as drug targets. Enzymes marked with orange asterisks are considered as potential drug targets. Folate cycle can provide cells with additional source of energy. Two molecules of NADPH are synthesized in cytoplasm in reactions catalyzed by DHFR (conversion of DHF to THF) and MTHFD1 (conversion of 5,10-methylenTHF to 5,10-methenylTHF), as well as in mitochondria by MTHFD2L (conversion of 5,10-methylenTHF to 5,10-methenyl THF). One molecules of NADPH is used by MTHFR which links Folate cycle to the Methionine cycle. Also, ATP can be synthesized during MTHFD1- (cytoplasm) or MTHFD1L-mediated (mitochondria) conversion of 10-formylTHF to THF.

Folates are referred to the family of B9 vitamins [33]. They are naturally present in different sources of food or can be synthesized chemically (e.g. folic acid) as dietary supplements. Folates function as carriers that distribute one-carbon groups from “inputs” to “outputs” (Figure 1 and 2). Once transported to the cell, the vitamin undergoes covalent modification by polyglutamination. It is further substituted by the one-carbon moiety in the N5 and/or N10 position at different oxidation levels: formate (10-formylTHF), formaldehyde (5,10-methyleneTHF), or methanol (5-methylTHF) [34].

As mentioned above, there are only two direct sources of 1C-groups in one-carbon metabolism – serine and glycine. Thus, the central reaction of the Folate cycle is conversion of serine to glycine by SHMT1 and SHMT2 enzymes. By transferring the 1C-group from serine and THF, this reaction generates 5,10-methyleneTHF – the first donor of one-carbon group in the folate cycle. Another source of 5,10-methyleneTHF comes from the enzymatic cleavage of glycine by an enzyme called glycine decarboxylase (GLDC), which resides in mitochondria.

In turn, 5,10-methyleneTHF can be used in three ways (Figure 2). First, it can serve as 1C-donor for the initial step of thymidylate biosynthesis, a reaction catalyzed by thymidylate synthase (TS). In this reaction 5,10-methyleneTHF provides one-carbon group for the pyrimidine biosynthesis and is oxidized into dihydrofolate (DHF). In the next reaction dihydrofolate reductase (DHFR) reduces DHF to THF enclosing this metabolic loop.

Second, 5,10-methyleneTHF can be used by a cytosolic enzyme Methylenetetrahydrofolate reductase 1 (MTHFD1), or mitochondrial tandem enzymes Methylenetetrahydrofolate reductases MTHFD2L/MTHFD2, to generate 10-formylTHF. 10-formylTHF is a 1C-donor for the two reactions of purine biosynthesis catalyzed by Trifunctional enzyme Phosphoribosylglycinamide Formyltransferase/ Synthetase/ Phosphoribosylaminoimidazole Synthetase (GART) and Bifunctional 5-Aminoimidazole-4-Carboxamide Ribonucleotide Formyltransferase/IMP Cyclohydrolase (ATIC), both of which in turn generate THF.

Third, 5,10-methyleneTHF is used by Methylentetrahydrofolatereductase (MTHFR) to generate methylTHF. The latter donates a methyl group to homocycteine resulting in the formation of methionine and THF. By this way the Folate cycle is coupled with Methionine cycle. Finally, THF is converted into 5,10-methyleneTHF by SHMT1 and SHMT2 thus enclosing the Folate cycle.

### Methionine cycle

**Figure 3:**
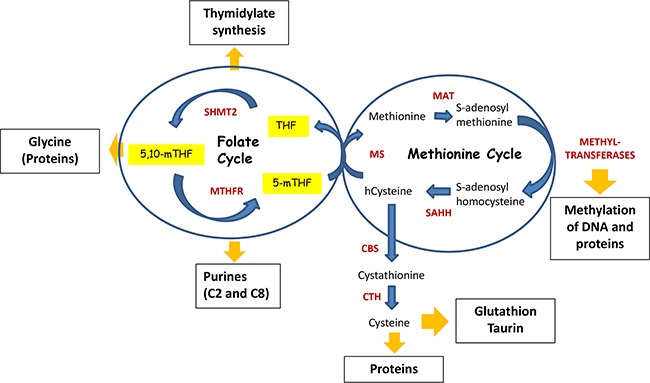
Folate cycle is coupled with Methionine cycle During Folate cycle MTHFR reduces 5,10-methyleneTHF to 5-methylTHF. Subsequently, 5-methylTHF donates its carbon group to convert homo-cysteine (hcystein) to methionine by methionine synthase (MS), hence initiating Methionine cycle. In turn, methionine is used by methionine adenosyltransferase (MAT) to generate S-adenosylmethionine (SAM) – the principal donor of methyl groups for DNA and proteins methylation. Thus, SAM is used by different methyltransferases, resulting in S-adenosylhomocysteine after its demethylation. Finally, S-adenosylhomocysteine hydrolase (SAHH) mediates deadenylation of S-adenosylhomocysteine to hcysteine, enclosing the methionine cycle. Homocysteine can be used by cystathionine synthase (CBS), which converts it to cystathionine. In turn, cystathionine is a substrate for cystathionine gamma-lyase (CTH), which uses it for synthesis of cysteine. Cysteine is required for the synthesis of proteins as well as for generation of taurine and glutathione, the latter is one of the critical molecules for redox homeostasis.

Another arm of the 1C-metabolic process is the methionine cycle (Figure 3). It starts with methionine synthesis from homocysteine and methylTHF catalyzed by methionine synthase (MS). Subsequently, methionine adenyltransferase (MAT) synthesizes SAM, the main donor of methyl groups in the cell. After demethylation, SAM is converted to S-adenosylhomocysteine (SAH). Finally, S-adenosyl homocysteine hydrolase (SAHH) mediates de-adenylation of SAHH resulting in homocysteine and full turn of the cycle.

## “OUTPUTS” OF ONE-CARBON METABOLISM

The Folate and Methionine cycles mediate redistribution of 1C-groups results in biosynthesis of a number of important compounds including nucleotides, several aminoacids, and GSH and SAM molecules that are critical for the maintenance of cell redox status and epigenetic homeostasis (Figure 1). All of these compounds are necessary for the rapid proliferation of cancer cells.

### The biosynthesis of nucleotides

One of the main outputs of one-carbon metabolism is biosynthesis of nucleotides. This is one of the molecular processes that constrains quickly proliferating cancer cells rate-limiting as it provides building blocks for DNA synthesis, purines and pyrimidines. The biosynthesis of both purine and pyrimidine (thymidylate) nucleotides requires cofactors generated through 1C-metabolism pathways.

### Pyrimidines

**Figure 4:**
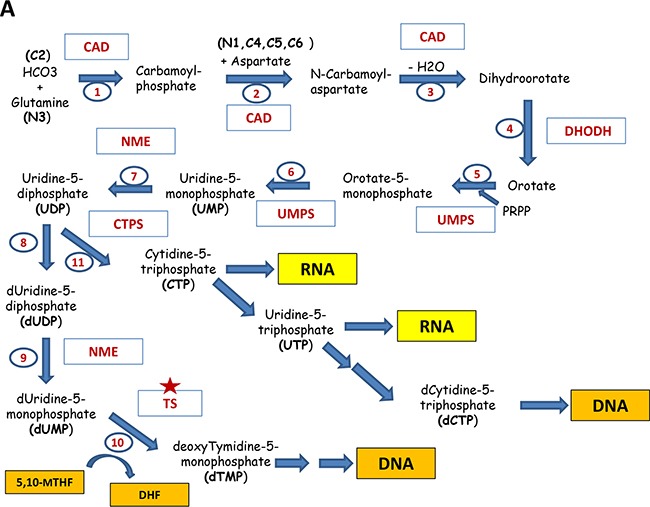

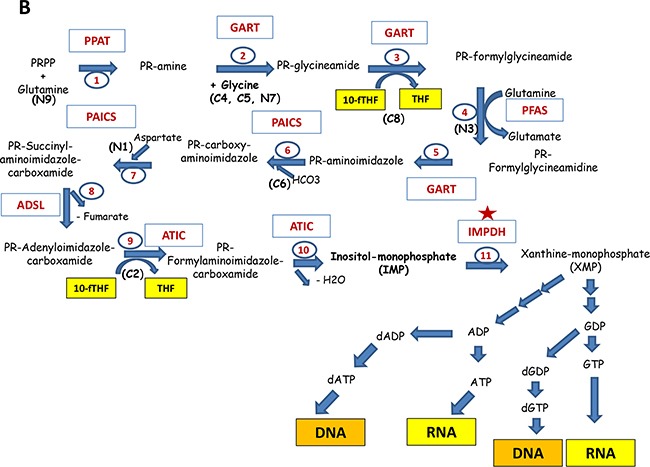
Major pathways of the nucleotide biosynthesis **A**. Shown are the reactions of the biosynthesis of pyrimidines: The participating enzymes are shown in red. The input compounds, intermediates and the resulting products are indicated (future positions of atoms in the nucleotide are indicated in brackets). Reactions are numbered in sequential order. Thus, reactions 1, 2 and 3 are catalyzed by tri-functional enzyme Carbamoyl Dehydrogenase (CAD); 4 – Dihydroorotate Dehydrogenase (DHODH);5 and 6 – bifunctional Uridine Monophosphate Synthetase (UMPS); 7,8,9 - Nucleoside Diphosphate Kinase (NME); 10 - Thymidylate synthase (TS), 11 - CTP Synthase (CTPS). Red asterisks indicate the enzymes that are currently being explored as drug targets. Enzymes marked with orange asterisks are considered as potential drug targets. **B**. The biosynthesis of purines. Abbreviations are the same as in part A.

The pyrimidine ring is composed of three fragments: C4 to C6 and N1 atoms are provided by aspartate, whereas C2 is derived from HCO3^−^, while N3 – from glutamine. The central precursor for generating pyrimidines is uridine-monophosphate (UMP). At first, the pyrimidine ring is constructed followed by conjugation with phospho-ribosyl-pyrophosphate (PRPP) (for details see Figure 4A). The synthesis of UMP does not require the 1C-cofactors. UMP through UDP can be than converted to either dUMP (for subsequent dTTP synthesis) or other nucleotides UTP, CTP and dCTP. The conversion of dUMP to dTMP is mediated by thymidylate synthase (TS) and requires 5,10-methylenTHF as 1C-donor. This reaction is very important for nucleotide biosynthesis and TS is a target for several approved drugs in cancer therapy (see below).

#### Purines

The purine ring is composed of different components (see Figure 4B): glycine, which is the most used precursor (donates C4, C5 and N7 atoms), HCO3^−^ - the donor of C6, glutamine (the donor of N3 and N9 atoms), aspartate (the donor for N1), and 1C-cofactor - N10-formylTHF which is a donor for C2 and C8. The central intermediate for purines is inosine-monophosphate (IMP). In contrast to pyrimidines, the purine synthesis starts with PRPP and subsequent step-by-step construction of the purine ring (for details see Figure 4). The IMP generation from PRPP needs five enzymes, three of which are multifunctional (GART, PAICS, ATIC). Reactions, catalyzed by GART and ATIC (the adding atoms C2 and C8 for purine ring) require N^10^-formylTHF produced by MTHFD1 and MTHFD2 during the folate cycle.

IMP can be converted in the two-steps reactions either into AMP or GMP. The gateway to guanine nucleotides is controlled by IMPDH, making it an “enzyme of consequence” for virtually every organism. This reaction is the first rate-limiting step in guanine nucleotide biosynthesis (Figure 4B).

Thus, the biosynthesis of nucleotides is critical for rapidly proliferating cancer cells to ensure the timely DNA replication and therefore represents a promising target for anticancer therapy (see below).

### SAM and epigenetic status

SAM, which is produced during the methionine cycle, is the principal donor of methyl groups [35]. It is required for methylation of histones and non-histone proteins, DNA, and other substrates. Methylation of both DNA and histones are well-known modulators of gene expression and are frequently altered in cancer [36, 37]. Tumors of different genesis usually display global hypomethylation and gene-specific hypermethylation [38].

Folate metabolism is closely linked to methionine cycle and, consequently, to DNA methylation (for more information see review of Crider et al. 2012 [39]). Indeed, THF donates one-carbons for homocysteine methylation resulting in synthesis of methionine [40]. By using colon cancer cell lines, researches have recently shown that serine contributed to SAM biosynthesis by providing one-carbon units to regenerate methionine in cells under methionine-depleted conditions [41]. Interestingly, they found that serine supported the methionine cycle in the presence and absence of methionine through *de novo* ATP synthesis. Cytoplasmic SHMT has also been shown to control the distribution of one-carbon units between the nucleotide synthesis and homocysteine methylation-mediated production of methionine [40, 42]. In addition, modulation of MTHFD1 expression affected the level of intracellular SAM [43]. Accordingly, several studies have shown that folate depletion was associated with the increased cancer incidence [44], coinciding with alterations in global methylation and gene expression [45–47]. There are several comprehensive reviews available that discuss the influence of folates on DNA methylation in cancer [39, 48, 49].

### Glutathione (GSH)

Serine can be condensed with homocysteine by the cystathionine synthase (CBS) to produce cystathionine, which is then cleaved by cystathionine lyase (CGL) to generate α-ketobutyrate and cysteine. This event is tightly connected with glutathione synthesis. Glutathione is a tripeptide composed of glutamic acid, cysteine and glycine. It plays the main role in the maintenance of the intracellular redox balance. The elevated levels of GSH are frequently observed in various cancers, which is associated with the increased resistance to chemotherapeutic drugs [50, 51].

During the Folate and Methionine cycles, 1C-groups are redistributed from serine and glycine to generate methionine and cysteine (in the cycle of Methionine), which, besides their role in the biosynthesis of SAM and glutathione, are necessary for the production of proteins.

### NADPH, ATP and energy

The well-known Warburg effect [52] postulates that cancer cells grown under conditions of normoxia have frequently intensified glycolysis. One explanation is that enhanced glycolysis is used to eliminate the lack of precursor metabolites which are required to rapidly proliferating cancer cells. But the alternative point of view suggests that glycolysis can also produce enough of energy by diverting its flux to other metabolic pathways including one-carbon metabolism [8]. According to this hypothesis, serine biosynthesis with one carbon catabolism and the glycine cleavage system represents a novel pathway for ATP and NADPH generation. Indeed, several reactions of one-carbon metabolism contribute to ATP and NADPH production (Figure 2).

Tedeschi with colleagues [9] showed that: 1. ATP production by cytosolic enzyme MTHFD1 is comparable to that of pyruvate kinase, 2. NADPH production by MTHFD2L is comparable to glutamine dehydrogenase, whereas the amount of its production by MTHFD1 is comparable with that of glucose-6-phosphate dehydrogenase. This production is balanced by purine, thymidylate, methionine and fatty acids biosynthesis.

In agreement with this, the treatment of cancer cells with methotrexate, an inhibitor of one-carbon metabolism, leads to the energy stress (increased AMP/ATP ration and activation of AMP kinase) and inhibition of purine and fatty acid synthesis [11].

Thus, the one-carbon metabolism not only facilitates anabolism by redistributing the 1C-units between the key enzymes, but also gives advantage to cancer cells by providing an additional source of energy generation.

## FOLATE TRANSPORT, MODIFICATIONS AND COMPARTMENTALIZATION

Mammalian cells are auxotrophic for folates due to the lack of enzymes responsible for their biosynthesis, therefore the cells require constitutive uptake of folate. Natural folates are metabolized in the mucosa of small intestine, generating its reduced form – tetrahydrofolate (THF), which can enter the metabolic cycle [53]. The predominant form of folate that takes place in the human body is 5-methylTHF (Figure 3), which is absorbed in intestine, circulates in the blood system and is delivered to all tissues [54].

### Folate transport across the cell membrane

There are three major ways for folate to entry through the cell membrane: 1) via reduced folate carrier (RFC); 2) proton-coupled folate transport (PCFT) and 3) folate receptors (FRs) (for comprehensive information see reviews [53, 55]. RFC (SLC19A1) is the main folate transporter in mammalian cells. It represents anionic pump transporting folates inside the cells with simultaneous counter transport of organic anions outside [56]. Its physiologic substrate is 5-methyl THF, the major circulating form of folate [57]. The specific feature of RFC mediated folate transport is a neutral pH optimum; its transporting properties significantly decrease at pH<7 [58]. Moreover, SLC19A1 is able to transport folate-mimicking antimetabolites – Methotrexate, Pralatrexate and Pemetrexed with micromolar affinities. It is ubiquitously expressed both in normal tissues and malignancies [56, 57, 59]. Thus, SLC19A1-mediated transport of antifolates into replicating cells of the bone marrow and intestinal tract is the basis for the major toxicities associated with these agents [60].

Another folate transporter is PCFT (SLC46A1), which is a low affinity heme transporter. It functions as a folate carrier in symport with protons flow down an electrochemical gradient concentration [61]. This occurs only at acidic pH<7. Thus, PCFT plays a pivotal role in intestinal absorption of dietary folates [62]. At the pH optimum, PCFT has a higher affinity for both natural folates and antifolates (0.2 to 5 μM). PCFT is also expressed ubiquitously but its functioning is restricted by the low pH optimum [53].

Because the efficient PCFT-mediated transport requires acidic pH, different tumors usually sustain anaerobic conditions to acidify their microenvironment. Therefore, it is tempting to speculate that specific targeting of tumors by newly designed antifolates can be increased should they have high affinity for PCFT and low for RFC [53].

The third folate transporting system is represented by the receptor-mediated endocytosis mechanism based on the high affinity and selective receptors, which are specific for reduced folates, as for the folic acid and some antifolates [63].

After folate binding to the FRs, the invagination of the cell membrane occurs followed by the formation of an endosome. Importantly, the release of folates is dependent on the endosome acidification [64]. There are evidences that PCFT participates in the low pH-dependent folate release from endosomes after their acidification [62].

There are three isoforms of human FRs encoded by different genes: FR α, β and γ [57]. It seems that these isoforms have different patterns of expression. For instance, FRα is expressed in epithelial tissues. On the contrary, FRβ is expressed in placenta and hematopoetic cells, namely of myelomonocytic origin [57]. The overexpression of FRα was shown in cervical and brain tumors, whereas FRβ was detected in acute and chronic myelogenous leukemia (AML and CML) [65–67] and in tumor-associated macrophages of various cancers [68–70]. Moreover, the level of FRα expression positively associates with tumor progression stages [71–73].

### Folate modifications

To become active, THF requires modification. Once inside the cell, folate molecules undergo polyglutamination by the enzyme folylpolyglutamate synthase (FPGS) adding up to 9 glutamate residues. So, polyglutamylated folates are predominating forms inside the cell [34]. Due to its polyanionic nature, this modification prevents export of the folate outside the cell [74]. The reaction reversed to polyglutamylation is catalyzed by lysosomal enzyme γ-glutamyl hydrolase (GGH). It catalyzes the hydrolysis of the γ-glutamyl tail of folate, as well as antifolate polyglutamates, which significantly decreases the activity of antifolates [75].

Besides naturally occurring folates, all currently approved antifolates also undergo polyglutamination, affecting their stability, affinity and even the target specificity.

### Folate compartmentalization

Inside the cell the folate pool is distributed between mitochondria, cytoplasm and nucleus (see comprehensive review [34]). Mitochondria accounts up to 40% of the intracellular folate [76], which is transported thitherward in the mono-glutamylated form by the SLC25A2 transporter [77]. Emerging evidence points to the very important role of SLC25A2 in cancer and suggests it as a potential anticancer drug target [78].

Mitochondrion contains its own system of glycine biosynthesis from serine generating 5,10-methyleneTHF. Also, the glycine cleavage system is located in mitochondria generating 5,10-methyleneTHF during the cleavage of glycine. Both of these processes require THF as a cofactor for SHMT2 and GLDC, respectively.

There are several observations that in different tumors and cancer cell lines predominantly mitochondrial enzymes involved in one-carbon metabolism are overexpressed, linking mitochondria folate metabolism to cancer progression. The expression of the mitochondrial glycine biosynthetic pathway strongly correlated with rates of proliferation across the panel of NCI-60 cancer cell lines [16]. A meta-analysis of the gene expression data showed that MTHFD2 and SHMT2 were at the top of consistently overexpressed mRNAs genome-wide across 19 different tumor types [79]. Moreover, the up-regulation of the mitochondrial folate and glycine–serine pathway is strongly correlated with increased sensitivity of NCI-60 cell lines to MTX [12], which demonstrates that mitochondrial pathways affect cell metabolic reprogramming and makes cancer cells dependent on one-carbon metabolism.

Overexpression of SHMT2 leads to transformation of NIH3T3 cells and makes them tumorigenic [17]. In contrast, SHMT2 knockdown decreases proliferation of fast-proliferating cancer cells by prolonging the G1 phase of cell cycle [16]. Lee with colleagues identified SHMT2 locus as a cancer-driving gene during mapping regions of recurrent amplification in a large collection of primary human cancers [80]. Furthermore, the study of breast cancer patients showed that SHMT2 was highly expressed in breast cancer cells, but not in their normal counterparts and the expression level of SHMT2 was positively correlated with breast cancer grade [81]. The meta-analysis of gene expression in different cancers has also shown that high level of SHMT2 expression associates with decreased patient's outcome [82], which suggests that up-regulation of SHMT2 is a common event common between different malignancies. All the data discussed here make SHMT2 a very attractive molecular target for future anti-cancer therapies.

As in the case of SHMT2, MTHFD2 is also a promising target for chemotherapy [11]. Knockdown of MTHFD2 decreases proliferation of cancer cell lines [79] and downregulates their migration and invasion [83, 84]. Besides, MTHFD2 has an important role in 1C-metabolism [85].

## REGULATION OF 1C-METABOLISM AND NUCLEOTIDE BIOSYNTHESIS IN CANCER CELLS

As discussed above, the one-carbon metabolism and nucleotide biosynthesis are critical for proliferation of cancer cells and therefore are frequently up-regulated in tumors. Yet the full picture of how these genes are regulated has only recently begun to emerge and is far from completeness. The regulation one-carbon metabolism and nucleotide biosynthesis occurs on several levels – by and at the levels of transcription, expression (microRNAs) and by diverting metabolic fluxes (see below and Figure 4).

### Regulation by the P53 family proteins

Importantly, enzymes involved in 1C-metabolism are tightly controlled not only on the protein level but also on the level of expression of the corresponding genes. Noteworthy, transcription factors that regulate expression of these genes belong to both tumor suppressors and oncogenes, thus forming a complex regulatory network. TP53 is the major tumor suppressor in mammals and its cellular level is tightly regulated by several ubiquitin-ligases, including Mdm2 [86, 87]. Ubiquitinylated p53 is targeted for degradation in 26S proteasomes [88]. Upon various forms of stress, including the metabolic one, p53 undergoes a cascade of phosphorylation-methylation-acetylation covalent modifications [89–91] that renders p53 inert to the proteolytic activity of proteasomes. Interestingly, upon stress proteasomes become covalently modified themselves resulting in attenuation of the proteolytic activity and binding RNA [91–93]. Counter-intuitively, p53 was shown to promote cell survival upon serine starvation by eliciting transient p21-dependent cell cycle arrest. This allowed cells to re-route depleted serine to glutathione synthesis, thus preserving cellular anti-oxidant potential [20]. Moreover, several studies have shown that p53 family proteins are able to induce the expression of glutaminase-2 (GLS2), which converts glutamine in glutamate thereby driving the serine biosynthetic pathway [94, 95]. Also, p53 was reported to increase glycolysis [96].

Besides p53, there are at least two ot her proteins in the family, p73 and p63, which also exert tumor suppression functions as transcription factors [97–99]. Again, similar to p53, p73 was shown to indirectly upregulate serine biosynthesis in cancer cells by promoting the expression of GLS2 [100]. In accordance with these results, TAp73 depletion completely abrogated cancer cell proliferation under conditions of serine/glycine-deprivation, supporting the pro-survival role of p73 in cancer cells under metabolic stress. Also, p73 was shown to upregulate glucose-6-phosphate dehydrogenase [101] thereby facilitating the pentose phosphate pathway and favoring nucleotide biosynthesis.

### Regulation on the level of microRNA

Recently, p53 was found to control gene expression by regulating micro-RNAs, small non-coding RNAs that target mRNAs of multiple genes [102, 103].

**Figure 5:**
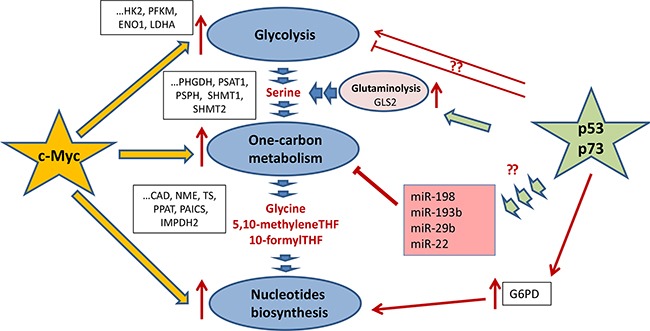
Regulation of one-carbon metabolism and nucleotide biosynthesis by p53 and c-Myc c-Myc and p53 (as well as its homologue p73) are the main trascription factors, which regulate glycolysis, one-carbon metabolism, and the nucleotide biosynthesis (shown as dark-blue ovals). c-Myc (denoted as a yellow star) is a master regulator of the key enzymes for both one-carbon metabolism and the nucleotide biosynthesis as well as its feeder pathway – glycolysis. The regulated genes are shown in black frames. Both p53 and p73 tumor suppressors (depicted as a green star) increase glutaminolysis (shown as a purple oval). They do it by activating the expression of GLS and hence enhancing the serine biosynthesis upon serine starvation. Also, p73 was shown to upregulate glucose-6-phosphate dehydrogenase thereby facilitating the pentose phosphate pathway and favoring the nucleotide biosynthesis. Also, p53 affects the expression of several micro-RNAs (shown in purple square), which target genes whose products are involved in the Folate and Methionine cycles.

In this regard, it is important to note that several genes involved in 1C-metabolism are regulated by micro-RNAs in a p53-dependent manner [104, 105], (Figure 5). For example, miR-198 attenuates expression of SHMT1, which is one of the key players in serine metabolism. Importantly, a search for new p53-regulated micro-RNAs has identified a p53 Response Element in the promoter of miR-198 gene [106]. Another critical enzyme of serine metabolism, SHMT2, was predicted to be the target of miR-193b [107]. Apparently, miR-193b is the target of p53, since the latter was shown to bind and activate the promoter of miR-193b in HCT116 cells treated with DNA damage [108]. Further, miR-22 which, together with miR-29b, is implicated in down-regulation of Methionine adenosyltransferase alpha1 (Mat1a) and Methylene tetrahydrofolate reductase (MTHFR) genes is also regulated by p53 [109]. MTHFR plays an important role in the 1C-metabolism linking the Folate and Methionine cycles [110]. Additional indirect evidence that signifies the importance of miR-22 in regulation of 1C-metabolism is the fact that miR-22 is significantly up-regulated in cells grown under low-folate conditions [105]. Despite the fact that more experimental work is required to decipher the role of p53-dependent micro-RNAs in regulation of metabolism, the already available data provide interesting therapeutic approaches that might be explored in future.

### Regulation by c-Myc

Another, perhaps the most critical regulator of metabolic pathways is the c-Myc oncogene. A number of studies demonstrated the importance of c-Myc in mediating glucose uptake, increased glycolysis, glutaminolysis and the fatty acids metabolism [111, 112].

Recent work has shown that c-Myc affects *de novo* serine synthesis during starvation of cancer cells [15]. The authors demonstrated that deprivation of glucose or glutamine, two major nutrition sources for cancer cells, led to the enhanced c-Myc expression, which in turn activated the expression of three key enzymes of serine synthesis, PHGDH, PSAT1 and PSPH (Figure 4).

In addition, c-Myc enhances expression of both SHMT1 and SHMT2 [113]. Several works have also shown that a set of enzymes involved in the nucleotide biosynthesis are transcriptional targets of c-Myc [114–116]. In the review by Lane and Fan [116] are summarized the predicted binding sites for different transcription factors, including c-Myc, in the promotors of all genes related to nucleotide biosynthesis pathways. It is likely that chromatin modifications are also involved in the regulation of metabolic genes. For example, the histone H3 lysine 9 (H3K9) methyltransferase G9A is required for the maintenance of genes involved in the serine biosynthesis pathway in an active state in response to serine deprivation [117]. However, it should be noted that for most of the transcription factors, except c-Myc, the experimental data are still lacking. Significantly, many tumors bear Myc amplification and/or overexpression [118] which could explain frequently observed up-regulation of one-carbon metabolism in different malignancies.

Another example of transcriptional regulation of metabolism is the regulation of DHFR by E2F1 [119]. Noteworthy, we have recently shown that the activity of E2F1 is critically dependent on the presence of another lysine methyltransferase, Set7/9 [90]. Our unpublished results suggest that Set7/9 is also involved in regulation of several metabolic genes (Shuvalov et al, unpublished).

### Regulation by metabolic fluxes

A mechanism regulating the flux of carbon sources into one-carbon metabolism is serine-mediated allosteric activation of pyruvate kinase (PKM2) [120, 121]. This enzyme mediates the last step of glycolysis by converting phosphoenolpyruvate (PEP) to pyruvate, which is then used by Pyruvate dehydrogenase complex converting it to acetyl-CoA. PKM2 is a splice variant of the *PKM* gene and is expressed preferentially in proliferating tissues. PKM2 has lower enzymatic activity than PKM1, hence it favors the accumulation of glycolytic intermediates due to reduced PEP conversion [122]. This, in turn, contributes to the elevation of the PPP level and serine synthesis. Thus, the regulatory loop can be envisioned as follows: an increase of serine levels promotes the activity of PKM2 by inducing allosteric changes in the enzyme, which results in reduced carbon flux into the serine biosynthesis pathway, and vice versa [120, 121].

## DIETARY EFFECTS CONNECTING ONE-CARBON METABOLISM AND CANCER

Diet is arguably one of the most important contributory factors to the development and progression of cancer [123]. The number of facts links the low sugar, low fat, low carbohydrate, and high protein diet and calorie restriction to both decreased incidence of cancer and reduced tumor growth [124–126].

The association between cancer progression and one-carbon metabolism was established by scientists in the last century. In 1945, Leuchtenberger and colleagues [127] reported that folic acid administration led to complete regressions of spontaneous breast cancers in 43 percent of mice. Based on this observation, Sydney Farber with colleagues [128] administrated folic acid to children with leukemia and observed that, contrary to the expectations, this supplement accelerated cancer progression.

In the following work, the same authors showed that folate antagonist aminopterin promoted complete remissions in children with acute leukemia, which gave rise to the antifolate-based cancer chemotherapy [129]. To date, a large amount of evidence demonstrates that the relation between folate consumption and the risk of cancer is very complicated and requires additional studies [130]. The colorectal cancer seems to be very controversial in particular [131].

Thus, it was concluded that folic acid fortification reduces the risk of certain childhood cancers in the offspring as well as prevents the development of cancers in normal tissues. On the other hand, folic acid supplementation and fortification may promote the progression of already existing pre-neoplastic and neoplastic lesions.

## PHARMACOLOGICAL TARGETING OF ONE-CARBON METABOLISM AND NUCLEOTIDE BIOSYNTHESIS

Since one-carbon metabolism is highly important for sustaining rapid proliferation of cancer cells, its inhibition primarily results in the downregulation of nucleotide biosynthesis and subsequent cell death. Thus, in general there are two overlapping ways for anti-metabolite therapy: inhibition of the folate cycle (indirect inhibition of nucleotide synthesis) and direct inhibition of the nucleotide biosynthesis.

### Antifolates

**Figure 6:**
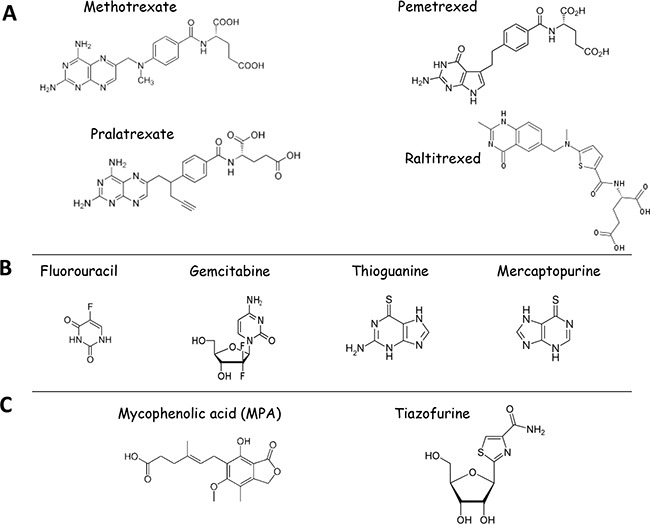
Structures of clinically-approved inhibitors of one-carbon metabolism and nucleotide biosynthesis used as anticancer therapeutics **A**. Shown are the structures of antifolates used in clinics. **B**. Structures of nucleoside analogs currently used in anti-cancer therapy: Inhibitors of nucleotide biosynthetic enzymes 5-Fluouracil (inhibitor of TS) and Gemcitabine (inhibitor of RNR), Antimetabolites - Thiopurines (Thioguanine and Mercaptopurine). **C**. Structures of IMPDH inhibitors currently undergoing clinical trials.

Antifolates denote a class of antimetabolite molecules, which are similar in structure to natural folates and thus compete with them for binding enzymes of the folate cycle and nucleotide biosynthesis (Figure 6A). Thus, antifolates favor the inhibition of nucleotide biosynthesis both directly and indirectly. Direct inhibition of the nucleotide biosynthesis by antifolates involves their physical binding to and inhibition of several enzymes of the nucleotide biosynthesis (e.g. TS, GART). Indirect inhibition is mediated by the antifolate-mediated blockage of the folate cycle (i.e. inhibition DHFR, which regenerates THF). By this way, antifolates prevent the generation of folates which are one-carbon donors for reactions of the nucleotide biosynthesis (5,10-methylenTHF and 10-formylTHF).

### Methotrexate

The inhibition of folate cycle is used as anticancer therapy since 1948 when Sydney Farber with colleagues applied aminopterin, the first antifolate, for the treatment of childhood ALL [129]. Soon after, the clinical application of aminopterin was replaced by methotrexate (MTX) which was more efficient and less toxic. Up to now, MTX is part of several poly-pharmacological schemes used for treatment of different malignancies (non-Hodgkin lymphoma, breast cancer, head and neck cancer, osteosarcoma, bladder cancer, colon cancer) as well as other non-cancer autoimmune diseases (rheumatoid arthritis, Crohn's disease, and psoriasis) [132].

The main target of MTX is DHFR. Whereas *in vitro* MTX can inhibit DHFR with Ki of 5 pM [133], in cell-based experiments the Ki of MTX was significantly higher (Ki ≥1 μM) [134]. This discrepancy can be explained by several reasons such as the efficacy of cellular import, post-translational modifications (polyglutamination), and fast efflux [74]. MTX is imported mainly by RFC [135] and PCFT [61] and a decrease in their expression is inversely associated with the increased resistance to this drug. The degree of polyglutamination positively correlates with MTX stability and its retention inside cells. Moreover, polyglutaminated MTX acquires ability to target also TS and ATIC, whereas it does not influence Ki of MTX towards DHFR. Finally, MTX is a substrate for ABC transporters (MRP1-5), which also have significant impact on the drug efficiency [132].

The SHMT2 and MTHFD2 genes are the major prognostic markers of susceptibility to MTX [12]. In addition, TYMS, DHFR and CTPS can also define the efficacy of the drug [136]. Thus, it seems that cells with elevated expression of these enzymes undergo metabolic reprogramming and exhibit intense one-carbon metabolism and, as a consequence, are sensitive to its inhibition.

The MTX treatment leads to the inhibition one-carbon metabolism which is primarily manifested in inhibition of the purine and glutathione biosynthesis, decreased protein and DNA methylation, impaired DNA synthesis and repair, activation of AMPK and, finally, cell cycle arrest or cell death [11]. Besides limited efficiency, MTX administration is frequently linked to nephrotoxicity, hepatotoxicity and neurological damage (memory loss). Therefore, several MTX derivatives with lower cytotoxicity have been developed and three of them were approved for clinical applications.

### Pralatrexate

Pralatrexate (Folotyn®) was specifically designed to improve cytotoxic properties of MTX. Substitution of the methyl group with the propargyl moiety at the N10 position significantly enhanced transport across cell membrane due to the increased affinity of pralatrexate to RFC [74]. This dramatically enhanced its anticancer activity in both *in vitro* and *in vivo* models [137, 138]. Importantly, this modification did not alter its affinity for DHFR [139]. Pralatrexate is approved for the treatment of patients with relapsed or refractory peripheral T-cell lymphoma (PTCL) http://www.rxlist.com/folotyn-drug/indications-dosage.htm

### Raltitrexed

Raltitrexed (Tomudex®; ZD1694) is an inhibitor of TS (Ki = 62 nM) [140]. It is transported into the cells by RFC and FR. Poluglutamylation of Raltitrexed results in significant increase of its affinity towards TS [74]. It was approved for treatment of colorectal, breast, gastroesophageal cancer and mesothelioma.

### Pemetrexed

Pemetrexed (Alimta®) is a ring substitution derivative of lometrexol, a non-approved inhibitor of GARFT. This modification alters the specificity of Pemetrexed primarily to TS [141]. Pemetrexed enters the cell mainly through PCFT and RFC and undergoes rapid polyglutamylation [61]. The advantage of Pemetrexed over MTX comes from a superior polyglutamination due to a 100-fold higher affinity for FPGS [142]. This results in better steady-stateof Pemetrexed over MTX. Moreover, polyglutamylation leads to increased affinity of Pemetrexed to TS (Ki = 1.3 nM for pentaglutamate form). It also inhibits GART (Ki = 65 nM), ATIC (Ki = 265 nM) and retains ability to inhibit DHFR (Ki = 7.2 nM) [74]. Therefore, Pemetrexed is called “multitargeted antifolate”. It is approved for treatment of non-squamous non-small cell lung cancer and malignant pleural mesothelioma in combination with cisplatin - (http://www.rxlist.com/alimta-drug/indications-dosage.htm)

In addition to these four approved MTX-based antifolates there is a number of yet non-approved compounds that differ in their status of polyglutamation. However, all of them target DHFR, TS and GART. For comprehensive information see the reviews [74, 132].

It should be noted that among approved one-carbon inhibitors most of them target predominantly DHFR and TS. However, as we have discussed above several other key enzymes of one-carbon metabolism and nucleotide biosynthesis including SHMT2, MTHFD2, MTHFD1, GLDC, GART and ATIC represent promising therapeutic targets.

In this respect, initial efforts to target GART go back to 1989. The first and one of the most promising GART inhibitors is Lometrexol [143]. As it has no effect on DHFR, TS and others thus making it a very specific inhibitor of purine nucleotide biosynthesis [74]. However, it has not been approved for clinical applications due to its high toxicity [144, 145]. Another potent inhibitor of GART is AG2034. It has similar to Lometrexol efficacy of GART inhibition (Ki = 28 nM) and demonstrated higher potency of tumor suppression [146]. However, similarly to Lometrexol, it showed high toxicity and severe side effects in clinical studies [147]. Taken these data together, this is a very intriguing question of whether it is possible to inhibit GART without a significant damage to healthy tissues. There are other new GART inhibitors including PY873, PY899, DIA [148, 149] and compound 12 [150] and it would be interesting to see the results of the clinical trials.

Mitochondrial enzymes SHMT2 and MTHFD2 are the key components of the folate cycle and as discussed previously, their deregulation plays a pivotal role in the cancer-related one-carbon metabolism. Also, it should be noted that these enzymes are preferentially expressed in rapidly proliferating (e.g. cancer) cells [16, 79] thus representing promising targets for antitumor therapy.

Finally, folate transporters, SL1C9A1 (plasma membrane) and SLC32A1 (mitochondrial) can also be considered as targets for inhibitors of folate metabolism [78].

## OTHER INHIBITORS OF METABOLISM

### Fluorouracil

Clinically approved TS inhibitor is Fluorouracil (5-FU) (Figure 6B). This is a nucleoside analog of uracil. The binding of 5-FU to TS inhibits the latter and blocks methylation that converts dUMP to dTMP. Moreover, inside cells 5-FU is metabolized to 5-fluorouridine which then incorporates into rRNA and interrupts protein translation [151]. 5-FU is approved for treatment breast, colorectal, oesophageal, stomach, pancreatic and skin and head and neck cancers. To enhance the efficiency of 5-FU binding to TS another drug, Leucovorin, is often used as an adjuvant therapy [151].

### Gemcitabine (Gemzar®)

Another clinically approved nucleoside analog is gemcitabine (Figure 6B). It targets ribonucleotide reductase (RNR) thus preventing the synthesis of deoxyribonucleotides required for DNA replication and repair [152]. Moreover, Gemcitabine incorporates into DNA and prevents synthesis of Cytidine thus terminating DNA synthesis [153]. Gemcitabine is approved for treatment of non-small cell lung cancer, pancreatic, ovarian, and breast cancers (http://www.rxlist.com/gemzar-drug/indications-dosage.htm).

### Thiopurines

Thiopurines belong to the class of anti-metabolite molecules and include 6-Thioguanine (6-TG), 6-Mercaptopurine (6-MP), and Azathioprine (Aza). All of them belong to the pro-drugs family and hence need a metabolic conversion to become active [154]. The metabolism of 6-TG and 6-MP culminates in the formation of 6-thiodGTP (6-TdGTP), a structural analog of guanine, which is a favorable substrate for DNA polymerases [155]. Once being incorporated into DNA during replication, 6-TdGTP causes cytotoxicity mediated by mismatch repair system (MMR) [156]. It has been proposed that post-replicative processing by MMR of aberrant base pairs containing 6-TG or O6-meG generates potentially lethal DNA lesions [157].

6-TG, 6-MP and Aza are preferentially used now as immunosuppressive drugs. But 6-TG (Tabloid®) and 6-MP (Purinethol®) (Figure 6B) have been applied for treatment of ALL and CML since 1950s till now [156], http://www.rxlist.com/tabloid-drug/indications-dosage.htm, http://www.rxlist.com/purinethol-drug/indications-dosage.htm].

### IMPDH2 inhibitors

As discussed above, the IMPDH-mediated oxidation of IMP to XMP is considered as the pivotal step in the biosynthesis of guanine nucleotide, whose pool controls cell proliferation and many other major cellular processes [158]. A decrease of the guanine nucleotide intracellular pool, which is a consequence of IMPDH inhibition, impairs the nucleic acid synthesis in proliferating cells. This makes IMPDH a crucial enzyme in cell proliferation and differentiation and consequently, an attractive anticancer target [159, 160]. Furthermore, in contrast to normal and quiescent cells, actively proliferating tissues, including tumors, usually overexpress IMPDH2, but not the IMPDH1 [160].

IMPDH2 is frequently overexpressed in different forms of leukemia [161], colorectal [162], prostate [18], bladder, and kidney cancers [163]. Moreover, there are several evidences that its expression is associated with highly invasive, metastatic cancers [18, 163] resistant to cisplatin and MTX [164, 165].

IMPDH is a known validated therapeutic target for treating several diseases including viral, microbial, and parasitic infections as well as immunosuppressive therapies. Several established inhibitors of IMPDH are widely used in clinics (e.g. ribavirin and CellCept®, a mycophenolic acid (MPA)) (Figure 6C). Recently, IMPDH has also become an attractive target for anticancer therapy. Several IMPDH inhibitors (Benzamide riboside, tiazofurine and MPA) (Figure 5) demonstrated high efficiency in preclinical studies [159, 165, 166, 167] and are currently undergoing clinical trials for the treatment of acute and chronic myelogenous leukemia (AML, CML). As IMPDH2 expression increases resistance to MTX, there is a possibility that application of IMPDH inhibitors can enhance the efficiency of MTX-related drugs [160]. There is also evidence that inhibition of IMPDH1 is sufficient to block angiogenesis [168].

## CONCLUDING REMARKS

One-carbon metabolism and nucleotide biosynthesis stand in one line with such cancer-related metabolic alterations as increased glycolysis, pentose-phosphate pathway and an acquired ability of *de novo* synthesis of fatty acids. According to the growing body of evidences, these metabolic features are common to different types of tumors and are considered now as one of the “hallmarks of cancer” [4, 6, 169]. They provide metabolic plasticity to cancer cells which has an impact on different features such as gene expression [170], epigenetic control [171] and drug resistance [172]. One carbon metabolism provides “building blocks” (nucleotides, certain aminoacids) as well as contributes to epigenetic (SAM for DNA and protein methylation) and redox (glutathione) homeostasis for rapidly proliferating cancer cells.

The high importance of one-carbon metabolism for cancer cells is reflected in more than 60 years application of its inhibitor (MTX) for a cancer treatment. But, one can notice, that despite the a relatively big number of enzyme operating in one-carbon metabolism and nucleotide biosynthesis, only a few of them are currently used as anticancer targets. They are only: DHFR, TS and RNR. Recently, IMPDH inhibitors became to be used and it will be interesting to see in next years if they are efficient to treat tumors.

Thus, an important question is whether other enzymes of one-carbon metabolism and nucleotide biosynthesis can be efficiently targeted by anticancer therapies without excessive toxicity to normal cells? For instance, as discussed above, there are number of potent GART inhibitors, but none of them has successfully passed through clinical trials due to their high toxicity. A related question is whether it is possible in general to target GART without cytotoxicity to normal cells?

As already discussed in previous sections, a number of very attractive and promising potential targets, including GLDC, SHMT2, and MTHFD2, have recently emerged. Their impact on cancer-related metabolism is very high; moreover, SHMT2 and MTHFD2 are preferentially expressed in quickly proliferating cells including cancer. The design of specific inhibitors is currently under way. It is likely that the combinatorial treatment with these novel inhibitors of 1C-metabolism together with currently used antifolate therapies should greatly improve the efficacy of chemotherapy by simultaneous targeting of one-carbon metabolism and nucleotide biosynthesis at different levels.

In spite the fact that the elevated metabolism and nucleotide biosynthesis are common features of different cancers [7, 12], the majority of the currently used specific inhibitors are efficient only in a few cancer types. A possible explanation to this phenomenon is that currently used antifolates are imported by cells with different efficacies and are processed with different kinetics. For instance, different expression levels of the key factors of folate import and metabolic processing can influence the outcome of antifolates treatment in various types of tumor. In this respect, micro-RNA profiling of cancer cells may predict the abundance of their targets, genes that code for metabolic enzymes. The latter, in turn, should determine the efficacy of highly specific drugs designed against these enzymes. Cancer cells, due to their genomic plasticity, can use parallel or bypassing metabolic pathways to escape the negative pressure conferred by specific drugs. Therefore, various combinatorial treatments that hit different aspects of cancer metabolism should be explored. Moreover, as cancer cells usually display complex metabolic alterations, the simultaneous targeting of several metabolic pathways (for instance, glucose and 1C-metabolism) seems to be an attractive strategy. A number of inhibitors of glucose metabolism are under clinical investigations [173, 174]. To make this therapy effective we need a better understanding of the metabolic flux in cancer cells [174]. Clearly, more pre-clinical and clinical studies are required to solve this important conundrum.

## Abbreviations

1C: One carbon
PHGDH: Phosphoglycerate dehydrogenase
PSAT1: Phosphoserine aminotransferase 1
PSPH: Phosphoserine phosphatase
SHMT2: Serine hydroxymethyltransferase 2, mitochondrial
MTHFD2: Bifunctional methylenetetrahydrofolate dehydrogenase/cyclohydrolase, mitochondrial
DHFR: Dihydrofolate reductase
MTHFR: Methylenetetrahydrofolate reductase
GLDC: Glycine dehydrogenase
IMPDH2: Inosine-5′-monophosphate dehydrogenase
TS: Thymidylate synthase
GART: Trifunctional enzyme Phosphoribosylglycinamide Formyltransferase/ Synthetase/ Phosphoribosylaminoimidazole Synthetase
ATIC: 5-Amino-4-imidazolecarboxamide ribonucleotide transformylase/inosine monophosphate cyclohydrolase
GCS: glycine cleavage system
THF: Tetrahydrofolate
5,10-mTHF: 5,10-methyleneTetrahydrofolate
5-mTHF: 5-methylTetrahydrofolate
10-fTHF: 10-formylTetrahydrofolate
DHF: dihydrofolate
SAM: S-Adenosyl-L-methionine
GSH: Glutathione
RFC: Reduced folate carrier
PCFT: Proton-coupled folate transporter
FR: Folate receptor
MTX: Methotrexate
5-FU: 5-Fluouracil
